# Occurrence of new or more severe headaches following COVID-19 is associated with markers of microglial activation and peripheral sensitization: results from a prospective cohort study

**DOI:** 10.1186/s10194-024-01810-6

**Published:** 2024-06-19

**Authors:** Johanna Ruhnau, Max Blücher, Susanne Bahlmann, Almut Zieme, Antje Vogelgesang, Anke Steinmetz, Robert Fleischmann

**Affiliations:** 1 Department of Neurology, University Medicine Greifswald, Greifswald, Germany; 2 Physical and Rehabilitation Medicine, Center for Orthopaedics, Trauma Surgery and Rehabilitation Medicine, University Medicine Greifswald, Greifswald, Germany

**Keywords:** Migraine, Headache, COVID-19, Sars-Cov-2, New daily persistent headache, Inflammation

## Abstract

**Background:**

New onset or worsening of a headache disorder substantially contributes to the disease burden of post-COVID-19. Its management poses a suitable means to enhance patients’ participation in professional, social, and personal activities. Unfortunately, the pathophysiology of post-COVID-19 headaches is poorly understood. This study aims to investigate the role of (neuro-) inflammatory mechanisms in order to guide the development of anti-inflammatory treatment strategies.

**Methods:**

We included patients from the interdisciplinary post-COVID-19 Rehabilitation Study (PoCoRe, *n* = 184 patients) run at a tertiary care university hospital, comprising patients with PCR-confirmed SARS-CoV-2 infection ≥ 6 weeks prior to their initial consultation. Patients reporting any headache since their infection were considered for this study (*n* = 93). These were interviewed and classified according to the International Classification of Headache Disorders, Third Edition (ICHD-3) by headache specialists. Patient sera were additionally analysed for levels of VILIP-1, MCP-1 (CCL2), sTREM-2, BDNF, TGF-ß1, VEGF, IL-6, sTREM-1, ß-NGF, IL-18, TNF-alpha, sRAGE, and CX3CL1 (Fractalkine). Markers of inflammation were compared between four groups of patients (none, unchanged, worsened, or new headache disorder).

**Results:**

Patients reported experiencing more severe headaches (*n* = 17), new onset headaches (*n* = 46), unchanged headaches (*n* = 18), and surprisingly, some patients denied having any headaches (*n* = 12) despite self-reports. Serum levels of CX3CL1 were increased in the worsened (2145 [811–4866] pg/ml) and new onset (1668 [0-7357] pg/ml) headache group as compared to patients with no (1129 [0-5379] pg/ml) or unchanged (1478 [346–4332] pg/ml) headaches. Other markers also differed between groups, but most significantly between patients with worsened (TGF-ß1: 60 [0-310] pg/ml, VEGF: 328 [86–842] pg/ml, ß-NGF: 6 [3–38] pg/ml) as compared to unchanged headaches (TGF-ß1: 29 [0–77] pg/ml, VEGF: 183 [72–380] pg/ml, ß-NGF: 3 [2–89] pg/ml). The results did not differ between headache phenotypes.

**Discussion:**

This study provides evidence that worsened or new headaches following COVID-19 are associated with pro-(neuro-)inflammatory profiles. This supports the use of anti-inflammatory treatment options in this population, especially in the subacute phase.

**Supplementary Information:**

The online version contains supplementary material available at 10.1186/s10194-024-01810-6.

## Background

The long-term consequences of COVID-19 (post-COVID-19) can be associated with a variety of symptoms, one of which is a new onset of headache or worsening of existing headache disorders [1–5]. The prevalence of headaches during acute COVID-19 infection varies widely, between 14% and 60% [4]. People with migraine have an increased risk of suffering headaches during acute COVID-19 infection [6], but this does not necessarily seem to lead to a higher incidence of headaches associated with post-COVID-19 [7]. Headaches after SARS-CoV-2 infection are usually reported to range between 10% and 19% [7–9], but there is also a meta-analysis with a frequency of 44% [10]. A case-control study also showed that 17.6% of migraine patients experienced a transformation of the headache into a chronic type [11]. The pathogenesis of the post-COVID-19 symptom is not yet precisely understood. Hypotheses include viral persistence, microthrombi, or ongoing immune system activation [12]. Recent studies show that the virus apparently cannot directly infect nerve cells [13], but that it can obviously lead to disruption of the blood-brain barrier [14]. This raises the question of the extent to which immunological mechanisms and, in particular, neuroinflammation are involved in post-COVID-19 headaches. This notion is supported by the finding of increased levels of TNF-alpha in new daily persistent headaches (NDPH) of other etiologies, yet the translation of these findings for post-COVID-19 headaches remains to be explored [15]. This being said, anti-inflammatory treatments, including intravenous corticosteroids and inhibitors of TNF-alpha (e.g., tetracycline and venlafaxine), have proven some efficacy in case series in NDPH [16]. The application of similar treatment strategies for the mitigation of post-COVID-19 headaches lacks evidence for the contribution of (neuro-)inflammatory mechanisms in their pathophysiology.

In this study, we explore the hypothesis that in individuals with post-COVID-19 headaches, which can manifest as either worsening of preexisting headache disorders or as new-onset headaches, neuroinflammatory markers will be elevated. For this purpose, a comprehensive biomarker profile is evaluated in a well-characterized prospective cohort of PCR-confirmed cases of post-COVID-19 headaches. Evidence in support of this hypothesis would suggest that neuroinflammation significantly contributes to the pathophysiology of post-COVID-19 headaches.

## Methods

### Ethical approval and study registration

Informed consent was obtained from all patients participating in the study. The study protocol was approved by the university ethics committee (No. BB 053/21) in accordance with the Declaration of Helsinki and the applicable legal regulations. The study was prospectively registered in the German Clinical Trials Registry (DRKS 00025007).

### Study design, participant selection, and headache classification

**Fig. 1:**
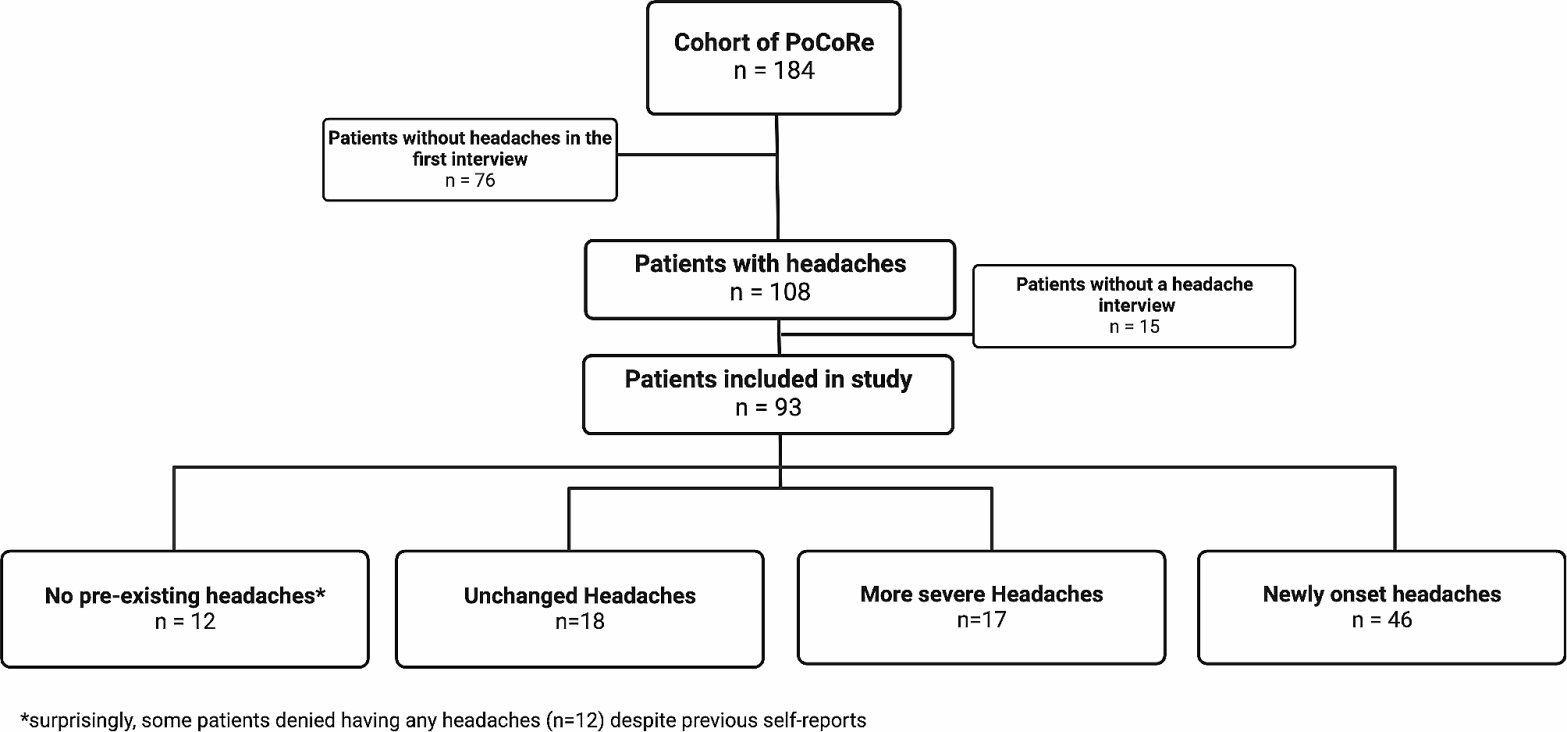
Flow chart of patients for analysing biomarker after COVID-19 infection. A total of 184 patients participated in the PoCoRe study conducted at University Medicine of Greifswald. To investigate headaches occurring after COVID-19 infection, 108 patients with a history of headaches were initially selected. They were then re-evaluated using standardized headache interview criteria based on the International Classification of Headache Disorders, 3rd edition, resulting in a subset of 93 patients. These patients experienced either unchanged preexisting headaches (*n* = 18), more severe pre-existing headaches (*n* = 17), or new headache disorders following COVID-19 (*n* = 46). Unexpectedly, some patients neither reported post-COVID-19 headaches nor had a history of headaches during the interviews, leading to the identification of a fourth group comprising patients without any headaches (*n* = 12)

This prospective cohort study was conducted with patients of the Post COVID-19 Rehabilitation Outpatient Clinic at the University Medicine in Greifswald, Germany, as part of the interdisciplinary Greifswald Post COVID-19 Rehabilitation Study and Research (PoCoRe). From April 2021 until 1 February 2022 184 patients were included in this cohort, which included patients ≥ 18 years of age and with any sequelae after PCR-confirmed SARS-CoV-2 infection ≥ 6 weeks prior to the initial consultation. Referrals to this outpatient clinic were mainly made by general practitioners, and therefore this cohort comprises predominantly patients who were not hospitalized during their COVID-19 disease. The study protocol contains a comprehensive and interdisciplinary assessment, including biosamples [17]. From this cohort, all patients who self-reported any headache were interviewed by a headache specialist to classify their headaches according to criteria in the International Classification of Headache Disorders, 3rd edition [18]. This included not only the definition of their phenotype but also the disentanglement of the worsening of a preexisting or occurrence of a new headache disorder. This resulted in the definition of three groups of patients, i.e., those suffering from an unchanged preexisting, more severe preexisting, or new headache disorder post-COVID-19. Some patients unexpectedly neither reported a headache post-COVID-19 nor a preexisting headache disorder in the interview, so a fourth group of patients without any headaches was established (compare Fig. 1).

### Laboratory analyses

All samples were acquired from patients of the PoCoRe study, University Medicine of Greifswald (Greifswald, Germany). Blood samples were stored at -80 °C until analysis [19]. The detection method employed in this study involved the use of a multiplex assay, specifically a commercially available assay known as the BioLegend® LEGENDplex™ Human Neuroinflammation Panel 1, accompanied by the LEGENDplex™ Data analysis software. This assay facilitated the simultaneous measurement of various serum biomarkers including VILIP-1: Visinin-like protein-1; MCP-1 (CCL2): Monocyte Chemoattractant Protein-1 (C-C motif chemokine ligand 2), sTREM-2: Soluble Triggering Receptor Expressed on Myeloid cells 2, BDNF: Brain-Derived Neurotrophic Factor; TGF-ß1 (free active): Transforming Growth Factor Beta 1 (free active); VEGF: Vascular Endothelial Growth Factor; IL-6: Interleukin-6; sTREM-1: Soluble Triggering Receptor Expressed on Myeloid cells 1; ß-NGF: Beta Nerve Growth Factor; IL-18: Interleukin-18; TNF-alpha: Tumor Necrosis Factor-alpha; sRAGE: Soluble Receptor for Advanced Glycation End-products; CX3CL1 (Fractalkine): C-X3-C Motif Chemokine Ligand 1 (Fractalkine). The measurements were conducted following the manufacturer’s instructions, albeit with a modified protocol involving halved consumables. Additionally, samples were re-measured if the coefficient of variation (CV) was equal to or greater than 30 in at least three analytes, ensuring accuracy and reliability in the data obtained.

### Data evaluation and statistics

Only patients who self-reported headaches (i.e., 93 out of 184 patients in the cohort) underwent investigations of biomarker profiles since we were primarily interested in understanding the difference between unchanged headaches and either new or more severe headache disorders post-COVID-19. Since there was an unexpected cohort of patients without any headache, comparisons of the biomarkers profiles of four different headache groups were carried out with SPSS (v28.0, IBM, Armonk, NY, USA) and GraphPad Software (version 7, La Jolla, CA). Exploratory analyses included the comparison of descriptive outcome data from the cohort (e.g., fatigue and depression) to understand the functional impact of post-COVID-19 headaches.

Normally distributed group data are presented as mean ± standard deviation; non-normally distributed data are presented as median and their interquartile range. Continuous data were analyzed for normal distribution using the Kolmogorov-Smirnov test. Inferential comparisons within and between group means were carried out using the Mann-Whitney U test for pairwise comparisons and the Kruskal-Wallis test for more than two groups. The analyses of medians were performed by Chi-square tests. Correlation analyses were performed by using Spearman or Pearson analysis based on data distribution. P-values below 0.05 were considered significant.

### Data availability

The data that supports the findings of this study is available from the corresponding author upon reasonable request.

## Results

### Demographics

This study examined the effect of COVID-19 on headache patterns using serum samples from 93 participants in total. Based on how the patients presented with their headaches, the patient population was split into four groups: those experiencing newly onset headaches after COVID-19 (*n* = 46), those with pre-existing headaches that were exacerbated after COVID-19 (*n* = 17), those with pre-existing headaches that remained constant after COVID-19 (*n* = 18), and those without headaches (*n* = 12) (compare Table 1).

Data analysis revealed that neither the prevalence of chronic comorbidities nor demographic variables significantly differed between the groups. However, significant variations were observed in headache type (*p* = 0.019), Functional Assessment Scale (FAS) scores (*p* = 0.007), Post-COVID Functional Status (PCFS) scores (*p* = 0.0007), and scores on the Patient Health Questionnaire-9 (PHQ-9) (*p* = 0.030) (compare Table 1). In terms of the interval between acute COVID-19 infection and the initial visit to the post-COVID-19 clinic, patients with unchanged headaches sought care sooner than those with more severe headaches (*p* = 0.0401). No other significant differences were observed between the groups.

**Table 1:** Patient characteristics: Overview of all patients, patients with no headaches, unchanged headaches, more severe headaches and new onset headaches for demographic data, headache subsets and co-morbidities, n = number, µ = mean, SD = standard deviation, p = *p*-value

	All patients	No headaches	Unchanged headaches	More severe headaches	New onset headaches	*p*
	*n* = 93	*n* = 12	*n* = 18	*n* = 17	*n* = 46	
**Demographic data**						
**Age [µ ± SD]**	49.74 ± 13.98	53.64 ± 15.17	48. 17 ± 12.03	54.76 ± 11.47	47.57 ± 14.56	0.23
**Sex. male [n (%)]**	25 (26.88)	5 (41.67)	7 (38.89)	2 (11.76)	11 (23.91)	0.18
**Sex. female [n (%)]**	68 (73.12)	7 (58.33)	11 (61.11)	15 (88.24)	35 (76.09)	0.18
**Headache days [µ ± SD]**	**9.59 ± 10.70**	**-**	**2.222 ± 3.098**	**14.94 ± 11.27**	**12.98 ± 10.81**	**< 0.0001**
**Intervall between acute COVID-19 infection and the first visit to the post-COVID-19 clinic [µ ± SD]**	**249.7 ± 166.0**	**268.7 ± 150.9**	**162.6 ± 99.53**	**290.6 ± 146.1**	**265.0 ± 190.3**	**0,0385**
Headache subsets						
**Tension-type headaches [n (%)]**	21 (22.58)	0 (0)	5 (27.78)	6 (35.29)	10 (21.74)	0.022
**Migraine [n (%)]**	33 (35.48)	0 (0)	7 (39.89)	11 (64.71)	15 (32.61)	0.022
**New daily persistent headache [n (%)]**	7 (7.53)	0 (0)	0 (0)	0 (0)	7 (15.22)	0.022
Others [n (%)] *	20 (21.51)	0 (0)	6 (33.33)	0 (0)	14 (30.43)	0.022
**Co-morbidities**						
**Hypertension [n (%)]**	27 (29.03)	6 (50.00)	4 (22.22)	5 (29.41)	12 (26.09)	0.34
**Diabetes mellitus [n (%)]**	11 (11.83)	1 (8.33)	1 (5.56)	3 (17.65)	6 (13.04)	0.7
**Chronic obstructive pulmonary disease [n (%)]**	12 (12.90)	3 (25.00)	3 (16.67)	1 (5.88)	5 (10.87)	0.44
**Depression [n (%)]**	15 (16.13)	1 (8.33)	2 (11.11)	3 (17.65)	9 (19.57)	0.73
**Rheumatic Disease [n (%)]**	4 (4.30)	1 (8.33)	1 (5.56)	0 (0)	2 (4.35)	0.73
**Active Cancer [n (%)]**	2 (2.15)	1 (8.33)	0 (0)	0 (0)	1 (2.17)	0.4
**Systemic immune suppression [n (%)]**	3 (3.23)	0 (0)	1 (5.56)	0 (0)	2 (4.35)	0.7
**Functional status**						
**Fatigue Assessment Scale (FAS) [µ ± SD]**	**36.25 ± 8.30**	**33.25 ± 8.86**	**31.22 ± 8.42**	**37.82 ± 6.75**	**38.41 ± 7.50**	**0.007**
**Post-COVID-19 Functional Status Scale (PCSF)** **[µ ± SD]**	**2.41 ± 0.61**	**2.00 ± 0.58**	**2.17 ± 0.60**	**2.29 ± 0.46**	**2.65 ± 0.56**	**0.0007**
**Montreal-Cognitive-Assessment (MOCA) [µ ± SD]**	25.64 ± 2.79	25.92 ± 2.66	26.59 ± 1.94	25.47 ± 2.55	25.27 ± 3.08	0.47
**Patient Health Questionnaire-9 (PHQ-9) [µ ± SD]**	**12.39 ± 5.85**	**11.27 ± 5.45**	**9.06 ± 4.10**	**13.53 ± 6.12**	**13.62 ± 5.88**	**0.030**

*Other headache subsets included: headache attributed to systemic viral infection (ICHD-3 9.2.2, x9), primary excercise headache (ICHD-3 4.2, x4), headache that could not been classified (x2), probable paroxysmal hemicrania (ICHD-3 3.5.2, x1), headache attributed to other non-infectious inflammatory intracranial disease (ICHD-3 7.3.3, x1), alcohol induced headache (ICHD-3 8.1.4, x1), headache attributed withdrawal from chronic use of other substance (ICHD-3 8.3.4, x1) and headache attributed to chronic ort recurring rhinosinusitis (ICDH-3 11.5.2, x1).

### Biomarkers for post-COVID-19 headaches

**Fig. 2:**
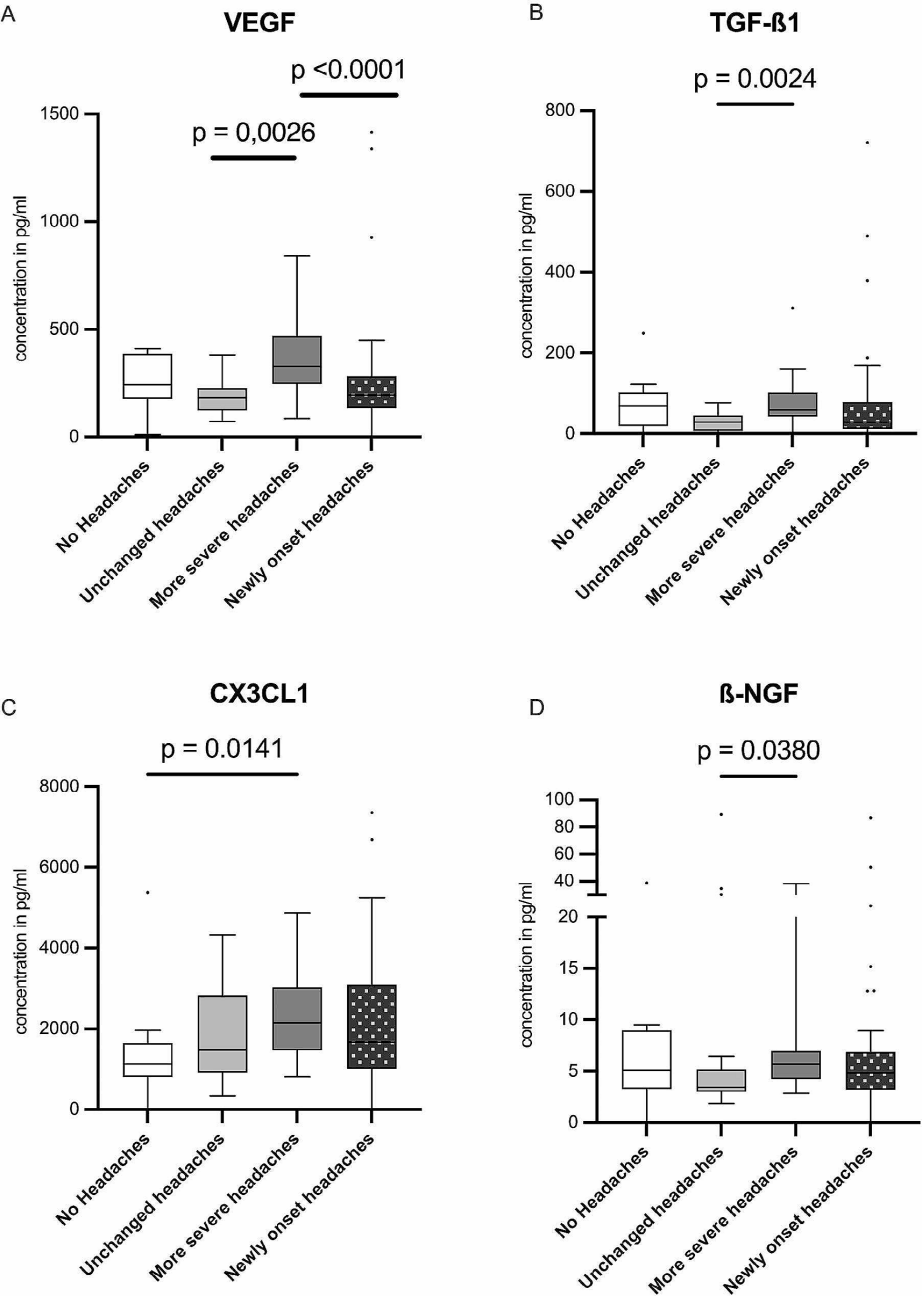
Comparison of VEGF (**A**), TGF-ß1 (**B**), CX3CL1 (**C**) and ß-NGF (**D**) concentrations for the 4 post-COVID headache groups: no headaches, unchanged headaches, more severe headaches, and newly onset headaches. Patient samples were analyzed for biomarker concentrations of VEGF (**A**), TGF-ß1 (**B**), CX3CL1 (**C**), and ß-NGF (**D**) across the four post-COVID headache groups: those with no headaches, unchanged headaches, more severe headaches, and newly onset headaches. Only biomarkers showing significant values < 0.05 in the global analysis of all biomarkers using the Chi-Square test are presented. Box and Whiskers plots are depicted

Compared to unchanged headaches, headaches that exacerbated after COVID-19 exhibited a significantly higher concentration of VEGF (*p* = 0.0022). On the other hand, headaches that started new after COVID-19 had a lower level of VEGF compared to those that were more severe post-COVID-19 (compare Table 2; Fig. 2A). TGF- β1 levels were notably increased in individuals experiencing more severe headaches as opposed to those with unchanged headaches (compare Table 2; Fig. 2B). A similar pattern was observed with CX3CL1, showing elevated levels, particularly in those with aggravated headaches after COVID-19 (compare Table 2; Fig. 2C). The average concentration of β-NGF among all studied patients was 17.83 pg/mL. Notably, only those with unchanged headaches had lower levels of this biomarker when compared to patients without headaches, those with more severe headaches, and those with newly onset headaches (compare Table 2; Fig. 2D).

In all patients, sTREM-2 (R² = 0.2281; *p* = 0.0279) and CX3CL1 (R² = 0.2438; *p* = 0.0185) were significantly correlated with the number of headache days. When divided into four headache subsets after COVID-19 infection, only BNDF was associated with a lower headache frequency (R² = -0.3008; *p* = 0.0422).

**Table 2:** Biomarkers for Post-Covid Headaches. Overview biomarkers of all patients, patients with no headaches, unchanged headaches, more severe headaches, and new onset. Data were analyzed for difference in median by Chi-square test. P-values are given for all biomarkers. Medians (Min- Max) are given

	All	No headaches	Unchanged headaches	More severe headaches	New onset headaches	Chi-Square	*p*
**VILIP-1**	994.75 (443.35–1546.15)	783.05 (0–2688.4)	670.92 (297–2549.45)	1056.9 (358.25 - 4464.65)	859.3 (0–6337.55)	6.428	0.093
**MCP-1**	235.29 (222.5–248.07)	366.8 (9.79–590.122)	297.364 (80.64–520.3)	364.51 (174.6–1312.0)	319.22 (0–708)	4.877	0.181
**sTREM-2**	5446.89 (2692.24–8201.53)	8422.74 (70.76–19782.60)	6977.22 (4371.60–12266.67)	9767.2 (4175.9–91514.75)	7238.69 (0–99043.53)	4.465	0.215
**BDNF**	25017.46 (24648.36–25386.56)	14874.17 (6.99–22277.11)	15930.60 (2065.7–30437.71)	15334.50 (433.66–27264.24)	12236.81 (0–570878.48)	4.556	0.207
**TGF- β 1**	**26.82** **(13.64–40.0)**	**68.9** **(0–249.22)**	**28.78** **(0–76.63)**	**59.8** **(0–310.96)**	**28.79** **(0–720.71)**	**7.871**	**0.049**
**VEGF**	**209.2** **(187.22–231.19)**	**242.65** **(10.36–411.86)**	**183.45** **(72.49–380.74)**	**328.32 (85.81–842.11)**	**194.04** **(0–1415.45)**	**15.212**	**0.002**
**IL-6**	20.67 (16.52–24.81)	21.64 (0–75.08)	17.27 (7.14–62.64)	25.19 (8.21–211.77)	20.18 (0–279.35)	6.428	0.093
**sTREM-1**	240.39 (84.42–396.36)	141.35 (0–715.09)	146.28 (24.96–725.65)	192.92 (51.41–1222.14)	214.00 (0–2054.66)	3.164	0.367
**β-NGF**	**17.83 (4.66–30.99)**	**5.06 (0.026–38.82)**	**3.45** **(1.83–89.24)**	**5.69** **(2.87–38.26)**	**4.838 (0.0085–86.90)**	**10.731**	**0.013**
**IL-18**	185.07 (156.29–213.86)	199.50 (0.04–760.93)	198.01 (35.97–487.51)	230.66 (57.71–1297.07)	219.99 (0–1925.43)	4.181	0.243
**TNF-alpha**	10.27 (6.18–14.36)	7.93 (0–51.18)	8.77 (1.18–42.29)	14.38 (4.41–59.15)	11.148 (0.083–65.40)	6.848	0.077
**sRAGE**	11280.55 (9404.15–13156.95)	2128.42 (219.38–18624.27)	8650.04 ( 611.38–32325.21)	2891.95 (680.36–33337.68)	6112.98 (195.41- 30145.02)	2.828	0.419
**CX3CL1**	**1140.08** **(986.9–1293.25)**	**1129.78 (0- 5379.15)**	**1478.08** **(346.45–4332.05)**	**2144.8 (811.1–4865.7)**	**1668.4 (0–7356.55)**	**11.064**	**0.011**

### Exploratory analyses of differences between headache phenotypes post-COVID-19

There was no significant difference between headache phenotypes based on ICHD-3 criteria (tension-type headache, migraine without and migraine with aura; all Kruskal-Wallis *p* > 0.05). Furthermore, there was no significant interaction between headache phenotypes and the four groups of post-COVID-19 headache status (data not shown).

## Discussion

Effective management of headaches in the context of COVID-19 and post- COVID-19 conditions remains a critical area for further research and clinical attention. Given the variety of headache manifestations related to COVID-19 and vaccination, individualized treatment strategies may be necessary [20]. In our study, TGF-β1, VEGF, CX3CL1 and β-NGF were elevated in patients with more severe headaches after COVID-19.

As shown by Silvestro et al., persistent headaches resembling migraines often occur after COVID-19, even in patients without a prior history of headaches, suggesting trigeminovascular activation due to inflammation [21]. The release of neuropeptides like Calcitonin Gene-Related Peptide (CGRP), potentially mediated by VEGF, leads to vasodilation and vascular leakage, while neurotrophins such as β-NGF modulate pain pathways and exacerbate migraine symptoms [22, 23]. The binary distinction between patients with unchanged/no headaches or changed/new headaches following COVID-19 exposure supports the general involvement of CX3CL1 and VEGF in the pathophysiology of post-COVID-19 headaches (Suppl. Figure 1). In accordance with our data, recent findings have demonstrated significantly increased concentrations of CX3CL1 in cerebrospinal fluid (CSF) both during headache episodes and interictally, compared to control groups [24]. Although the authors did not observe alterations in serum as identified in our post-COVID analysis, this discrepancy may be attributed to different mechanisms in post-COVID-19 pathophysiology. Moreover, patients with tension-type headaches are known to exhibit higher levels of TGF-β1 compared to healthy controls, with elevated TGF-β1 levels in the CSF of patients with both episodic tension-type headache and migraine without aura [25, 26]. These findings suggest that different compartments may exhibit distinct inflammatory responses associated with different headache phenomena. Therefore, further studies are necessary to delineate these differences and identify sufficient biomarkers. Furthermore, IL-6 was elevated in the group with more severe or newly onset headache status (Suppl. Figure 1). Interleukin-6 (IL-6), a pro-inflammatory cytokine, has been extensively studied in the context of COVID-19 due to its role in the cytokine storm syndrome, which could be linked to the pathogenesis of changed headaches through systemic inflammation [27]. The increased concentrations of IL-6 (Suppl. Figure 1) in new or more severe headaches compared to no or unchanged headaches in our data may reflect a heightened inflammatory state or a specific immune response in these individuals, while the increase in β-NGF in more severe headache patients (Fig. 1) suggests alterations in neurotrophic support or modulation of pain pathways. These findings are in line with the known roles of NGF in pain perception and neuroinflammation, suggesting a complex interplay between neurotrophic factors and headache severity. Although, in contrast to our study, the data on β-NGF were analysed in cerebrospinal fluid [28].

Independent of the neuro-inflammatory markers, we have classified patient-reported headaches associated with post-COVID-19 syndrome with a standardized headache assessment according to ICHD-3. At their first presentation, 56.6% of patients in the cohort reported headaches [29]. Now our stratification shows that as many as 6.5% had no headache diagnosis and 9.8% reported an unchanged pre-existing headache. The finding that 25% of patients present with new onset of daily headache in association with post-COVID-19 syndrome is consistent with recently published data [30]. Furthermore, the four headache groups also differed significantly in the Functional Assessment Scale (FAS); Patient Health Questionnaire-9 (PHQ-9) and Post-COVID Fatigue Scale (PCFS). The two groups with post-COVID-19 syndrome-associated headaches each show significantly higher values in these assessments than patients with an unchanged headache or without a headache diagnosis. Patients with a new onset of daily headache reported the highest values in each score. The post-COVID-19 syndrome associated headache types in particular also indicate a higher degree of fatigue (severe fatigue ≥ 35 points). The latest findings demonstrate that higher fatigue and depression scores in combination with post-COVID-19 syndrome-associated headaches are of great relevance concerning post-COVID-19 syndrome course and outcome [31]. In a longitudinal analysis of the German population-based COVIDOM/NAPKON-POP cohort, Hartung et al. showed that post-COVID-19 syndrome patients with fatigue and higher depression scores and/or headaches had a significantly higher risk of not recovering [32]. This emphasizes the need to precisely differentiate post-COVID-19 syndrome -associated headaches in their entity according to ICHD-3 as well as immunologically in order to develop effective treatment approaches.

### Limitations

This study only included patients that suffered some sort of impairment following COVID-19, which renders the possibility of a selection bias. Patients without any symptoms were unlikely to be referred to the post-COVID-19 outpatient service. Nonetheless, we found distinct immunological profiles among the four groups of post- COVID-19 headaches, and these should be considered specific irrespective of the selection bias. On the other hand, we cannot exclude the possibility that biomarker profiles in the group without headache are affected by the presence of long-COVID-19. Yet, given the vast prevalence of people having an, potentially unnoticed, infection with Sars-COV-2 renders future studies with an unaffected control group difficult. However, patients without long-COVID-19 would pose another suitable control group to validate our findings.

Another limitation is that patients were included several weeks following the infection but not necessarily in the chronic stage of their headache disorder. It thus remains unclear how the headache may have developed several months later. This being said, it is possible that identified (neuro-)inflammatory mechanisms rather contribute to the occurrence of worse or new headaches, but that other mechanisms (like acute infection after COVID-19 or other comorbidities) such as central sensitization contribute to persistent headache disorders following COVID-19 [33]. Furthermore, our study included a limited sample size as it was designed to explore the hypothesis that systemic and neuroinflammatory mechanisms are involved in the pathophysiology of COVID-19 headaches. While it is possible to apply corrections for multiple testing, particularly given our small sample size, such adjustments could obscure exploratory effects. Thus findings should be confirmed through future prospective studies. These studies should also evaluate the severity of headaches (i.e. intensity, number of acute medication days per month).

## Conclusion

To our knowledge this is the first study to provide evidence for distinct biomarker profiles in patients with a worsening of a preexisting or new onset of a headache disorder post-COVID-19. Increased levels of markers of systemic inflammation, neuroinflammation, and peripheral neuronal sensitization indicate that COVID-19 leads to an unspecific immunological response that affects neuronal structures involved in processing pain. These findings support the use of anti-inflammatory treatment strategies for the treatment of post-COVID-19 headaches in the subacute stage several weeks after the initial infection.

### Electronic supplementary material

Below is the link to the electronic supplementary material.

Supplementary Material 1: Suppl. Figure 1: Comparison of VEGF (A), IL-6 (B) CX3CL1 (C) and ß-NGF (D) concentrations for the 4 post-COVID headache groups: no/unchanged headaches, more severe headaches/newly onset headaches. Patient samples were analyzed for biomarker concentrations of VEGF (A), TGF-ß1 (B), CX3CL1 (C), and ß-NGF (D) across the two post-COVID headache groups: those with no headaches/unchanged headaches and those with more severe headaches/newly onset headaches. Only biomarkers showing significant values < 0.05 in the global analysis of all biomarkers using the t-test are presented. Box and Whiskers plots are depicted.

## Data Availability

The data that support the findings of this study are available from the corresponding author, RF, upon reasonable request.

## Abbreviations

VILIP-1: Visinin-like protein 1
MCP-1 (CCL2): Monocyte chemoattractant protein-1 (Chemokine (C-C motif) ligand 2)
sTREM-2: Soluble triggering receptor expressed on myeloid cells 2
BDNF: Brain-derived neurotrophic factor
TGF-ß1: Transforming growth factor beta 1
VEGF: Vascular endothelial growth factor
IL-6: Interleukin 6
sTREM-1: Soluble triggering receptor expressed on myeloid cells 1
ß-NGF: Beta-nerve growth factor
IL-18: Interleukin 18
TNF-alpha: Tumor necrosis factor alpha
sRAGE: Soluble receptor for advanced glycation end-products
COVID-19: Coronavirus Disease 2019
CNS: Central Nervous System
TRPV1: Transient Receptor Potential Vanilloid 1
CGRP: Calcitonin Gene-Related Peptide
BDNF: Brain-Derived Neurotrophic Factor
FAS: Functional Assessment Scale
PHQ-9: Patient Health Questionnaire-9
PCFS: Post-COVID Fatigue Scale
PoCoRe: Post COVID-19 Rehabilitation Study and Research (Greifswald)
NDPH: New daily persistent headaches
