# Supplementary figures and images for: Occurrence of new or more severe headaches following COVID-19 is associated with markers of microglial activation and peripheral sensitization: results from a prospective cohort study
Source: J Headache Pain. 2024 Jun 19;25(1):101. doi: 10.1186/s10194-024-01810-6 (PMC11186149; doi:10.1186/s10194-024-01810-6)

A

**VEGF** $p = 0.041$ 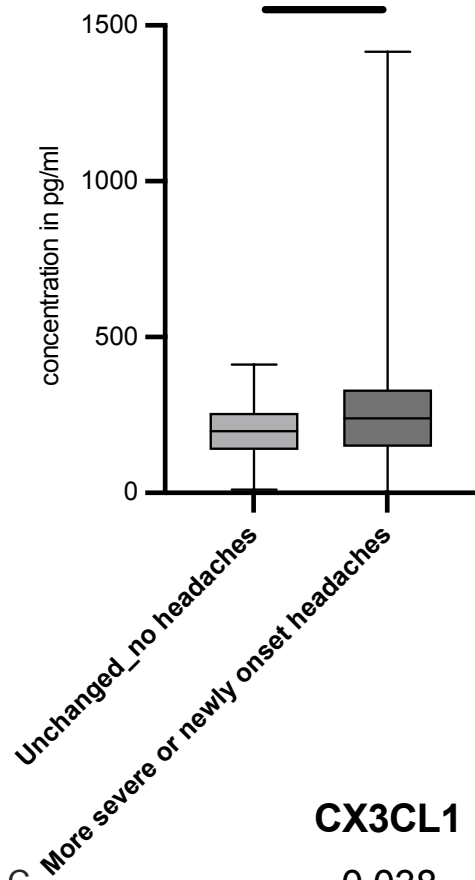

B

**IL-6**

0.040

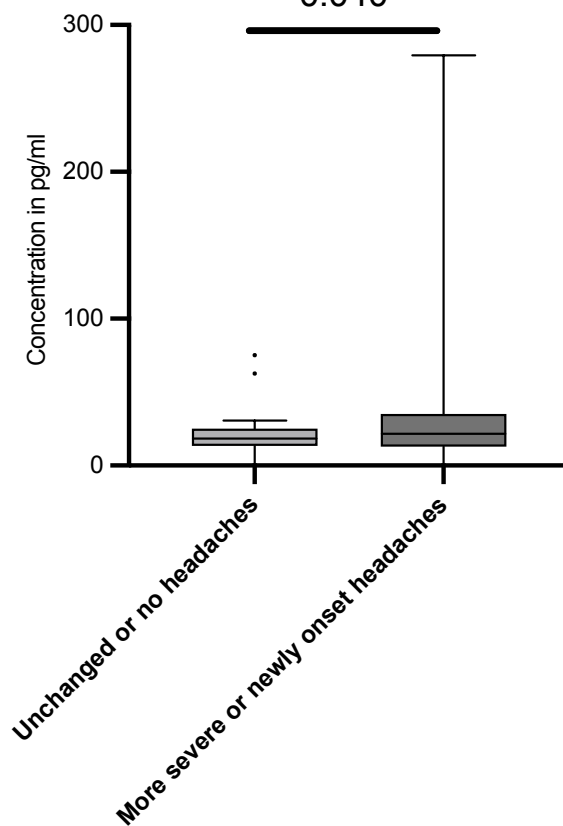

C

**CX3CL1**

0.038

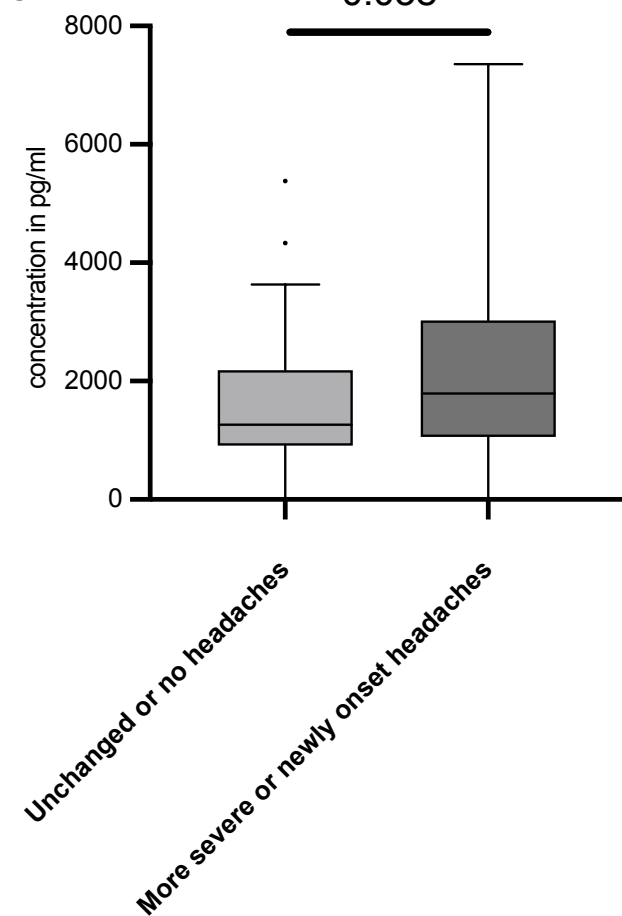

D

 **$\beta$ -NGF**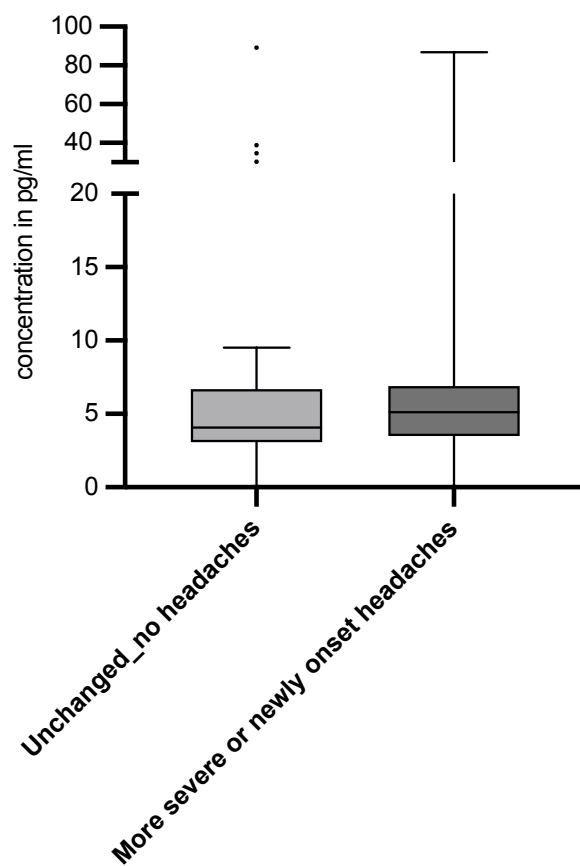

Supplement: Supplementary file 1 — Supplementary Material 1: Suppl. Figure 1: Comparison of VEGF (A), IL-6 (B) CX3CL1 (C) and ß-NGF (D) concentrations for the 4 post-COVID headache groups: no/unchanged headaches, more severe headaches/newly onset headaches. Patient samples were analyzed for biomarker concentrations of VEGF (A), TGF-ß1 (B), CX3CL1 (C), and ß-NGF (D) across the two post-COVID headache groups: those with no headaches/unchanged headaches and those with more severe headaches/newly onset headaches. Only biomarkers showing significant values < 0.05 in the global analysis of all biomarkers using the t-test are presented. Box and Whiskers plots are depicted. [file 10194_2024_1810_MOESM1_ESM.pdf]
